# A Rare Case of Pseudoangiomatous Stromal Hyperplasia in a Male Patient

**DOI:** 10.5334/jbsr.3356

**Published:** 2023-11-02

**Authors:** Qun Xia Xu, Johan Bafort, Jan Decloedt

**Affiliations:** 1 Department of Radiology, AZ Sint Blasius, Dendermonde, Belgium; 2 Breast Unit, AZ Sint Blasius, Dendermonde, Belgium

**Keywords:** PASH, breast, gynecomastia vera, anastomosing slit-like spaces, ultrasound

## Abstract

**Teaching Point:** Pseudoangiomatous stromal hyperplasia (PASH) is a rare benign breast condition that can mimic the appearance of breast cancer on imaging studies.

## Case History

**Figure 1:**
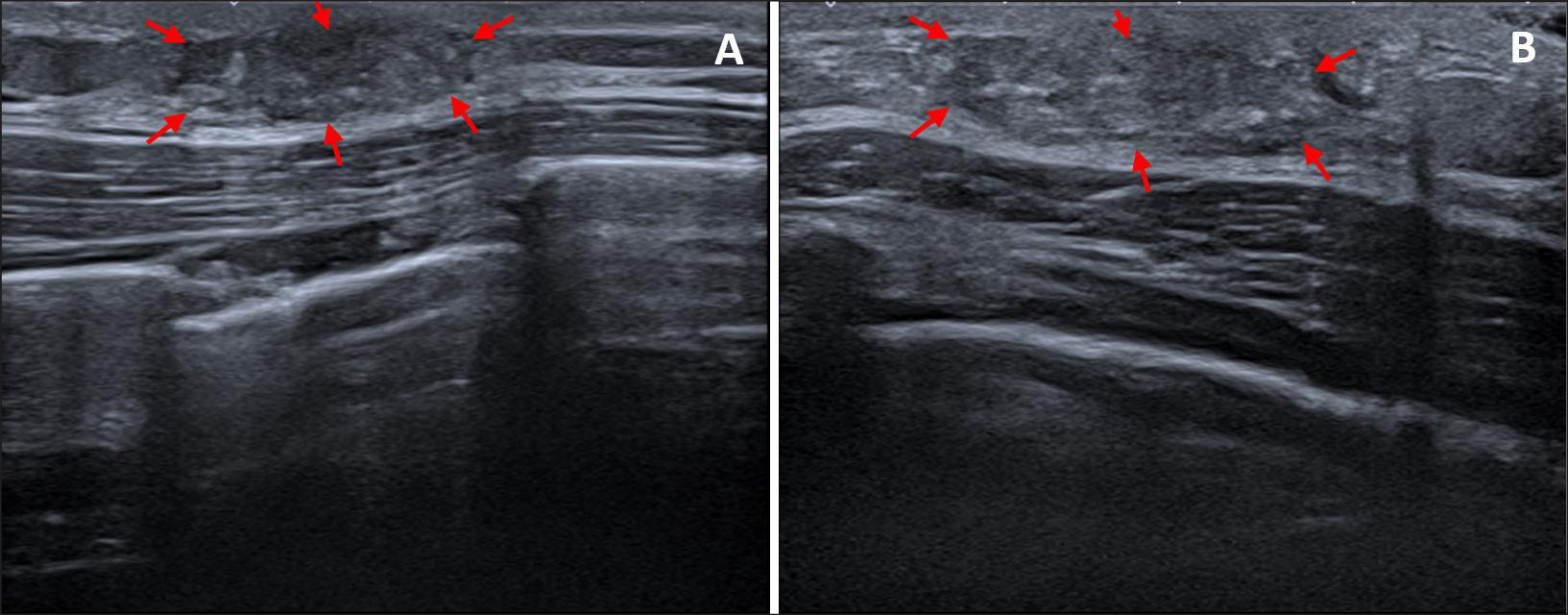


**Figure 2:**
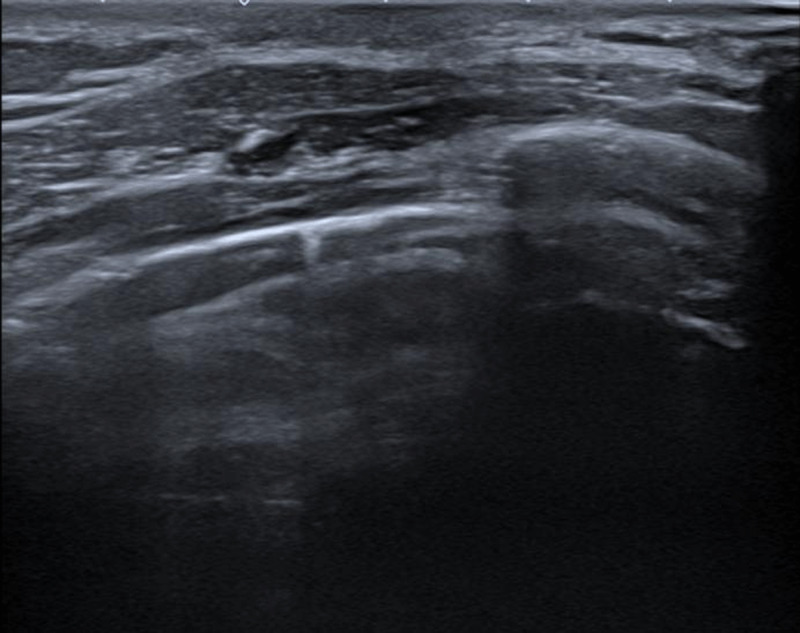


**Figure 3:**
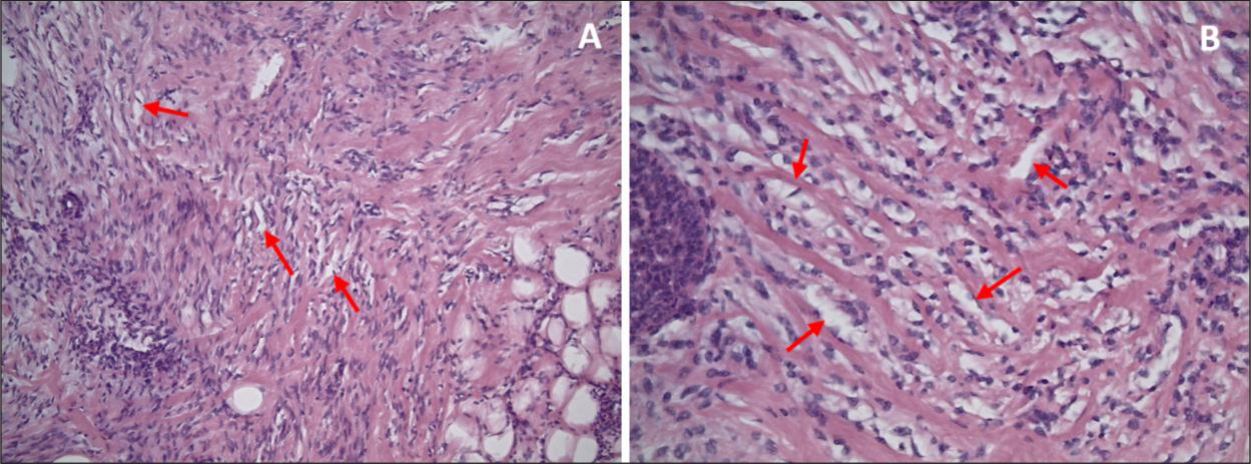


A 21-year-old male patient presented with a palpable nodule in the right breast for three weeks. There was no redness or pus, and he occasionally experienced pain. Clinical examination revealed a hard and mobile retro-areolar nodular structure. The patient’s medical and family history were unremarkable. Ultrasound revealed a hypoechoic circumscribed solid lesion retro-areolar, measuring 33×25×6 mm (LL×CC×AP), superficial to and with long axis parallel to the pectoralis muscle (Figure 1A–B, red arrows). As the lesion did not appear typical of gynecomastia vera, the patient agreed to perform an ultrasound-guided core needle biopsy as part of the triple assessment. The left breast appeared unremarkable (Figure 2). Histopathological examination showed an increase in ducts, which were lined by epithelial and myoepithelial cells without cytonuclear atypia. The stroma was rich in cells and demonstrated stromal hyperplasia with slit-like spaces (Figure 3A–B, red arrows), surrounded by fibroblasts. There were no signs of malignancy.

## Comments

Pseudoangiomatous stromal hyperplasia (PASH) of the breast is a benign mesenchymal proliferative condition. Histologically characterized by a network of anastomosing slit-like spaces in a background of dense, collagenous proliferating breast stroma [1].

A proposed etiology suggests that the proliferation of myofibroblasts is due to an abnormal response to estrogen and progesterone. PASH predominantly affects premenopausal or perimenopausal women and postmenopausal women on hormone replacement therapy [1]. It can occur rarely in men.

PASH can present as a breast lump, which may mimic breast cancer. On ultrasound, it usually appears as a well-defined hypoechoic solid mass. Suspicious features, such as architectural distortion and spiculated margins, are exceptionally rare [1]. Gynecomastia vera, fibroadenoma, lipoma, hematoma, breast cancer (ductal carcinoma) or angiosarcoma can be considered in the differential diagnosis.

In this rare case, PASH was found in a 21-year-old man with no significant medical history and no evidence of hormone therapy or steroid abuse. Distinguishing PASH from breast cancer can be challenging. Malignant transformation of PASH is extremely rare. On magnetic resonance imaging (MRI), tumorous PASH may appear as a well-defined lesion showing gradual progressive enhancement on post-contrast sequences, mimicking fibroadenoma. A definitive diagnosis needs to be established through biopsy to rule out malignancy [1].

In most cases, PASH does not require specific treatment. Serial imaging follow-up for a few years can be considered for cases that are small, incidentally discovered, or asymptomatic. Surgical excision is usually reserved for large symptomatic lesions with pain or enlargement [1]. In this case the patient chose a conservative method without surgical excision.
